# Mechanical and Thermal Dehydrogenation of the Mechano-Chemically Synthesized Calcium Alanate (Ca(AlH_4_)_2_) and Lithium Chloride (LiCl) Composite

**DOI:** 10.3390/ma8063479

**Published:** 2015-06-12

**Authors:** Robert A. Varin, Ewelina E. Kościuczyk, Tomasz Czujko

**Affiliations:** 1 Department of Mechanical and Mechatronics Engineering, University of Waterloo, 200 University Ave., Waterloo, ON N2L 3G1, Canada; 2 Department of Advanced Materials and Technologies, Military University of Technology, PL-00-908 Warsaw, ul. S. Kaliskiego 2, Poland; E-Mails: ekosciuczyk@wat.edu.pl (E.E.K.); tczujko@wat.edu.pl (T.C.)

**Keywords:** hydrogen generation, mechano-chemical synthesis, (Ca(AlH_4_)_2_+2LiCl) composite, mechanical and thermal dehydrogenation

## Abstract

LiAlH_4_ and CaCl_2_ were employed for mechano-chemical activation synthesis (MCAS) of Ca(AlH_4_)_2_ and LiCl hydride composite. After short ball milling time, their X-ray diffraction (XRD) peaks are clearly observed. After ball milling for a longer duration than 0.5 h, the CaAlH_5_ diffraction peaks are observed which indicates that Ca(AlH_4_)_2_ starts decomposing during ball milling into CaAlH_5_+Al+1.5H_2_. It is estimated that less than 1 wt % H_2_ was mechanically dehydrogenated in association with decomposition reaction. After 2.5 h of ball milling, no Ca(AlH_4_)_2_ diffraction peaks were observed on XRD patterns which suggests that Ca(AlH_4_)_2_ was decomposed. Thermal behavior of ball milled powders, which was investigated by thermal gravimetric analysis (TGA) and differential scanning calorimetry (DSC), indicates that a certain fraction of Ca(AlH_4_)_2_ could have been disordered/amorphized during ball milling being undetectable by XRD. The apparent activation energy for the decomposition of Ca(AlH_4_)_2_ and CaAlH_5_ equals 135 kJ/mol and 183 kJ/mol, respectively.

## 1. Introduction

The worldwide acceptance of the Hydrogen Economy would lead to a gradual elimination of the present fossil fuels-based economy and make a decisive turn to the economy based on renewable and clean resources [1,2]. The backbone of the Hydrogen Economy is a wide usage of fuel cells (FC) where hydrogen gas (H_2_), in contact with oxygen (O_2_), is converted into an electrical energy. An interrupted supply of H_2_ to fuel cells requires an efficient and inexpensive H_2_ storage or generation system. There are three possible storage systems for supplying H_2_ to a FC stack: gaseous H_2_, liquid H_2_ or solid metal/nonmetal hydrides [3,4]. For mass transportation by using automobiles, solid state hydrogen storage in hydrides has certain advantages over gas and liquid because most solid hydrides exhibit a higher H_2_ volumetric density than gas or liquid storage, and they don’t have serious safety problems such as a very high pressure of 70 MPa for H_2_ gas or large thermal losses for liquid H_2_ which requires a formidable insulation and an open storage system [3,4]. Moreover, both H_2_ gas and liquid storage suffer from substantial pressurization or liquefaction costs.

However, solid-state H_2_ storage in hydrides has its own drawbacks. First, the most common, high power density Proton Exchange Membrane fuel cell (PEM FC) (sometimes also named a Polymer Electrolyte Fuel Cell (PEFC)) stack generates the quantity of waste heat which is able to rise coolant temperature to barely 70–80 °C. Second, a PEM FC stack operates at H_2_ fuel pressure slightly above 1 bar (roughly 1.1–3.0 bar). Third, the US Department of Energy target for driving a fuel cell vehicle for 300 miles is 5.5 wt % H_2_ for the year 2017 for the entire fuel system [5], which requires at least about 11 wt % H_2_ capacity for the desorbing material. The Ultimate D.O.E. (Department of Energy) target is 7.5 wt % H_2_ which requires, at least, a whopping 15 wt % H_2_ capacity for the desorbing material.

In addition, an automotive H_2_ storage system must be reversible “on board”. So far, there is no solid hydride that can desorb under roughly 1.0–3.0 bar H_2_, at low temperatures, not exceeding the waste heat temperature of a FC stack (70–80 °C), which can be used for heating a hydride storage tank, has a capacity at least 11 wt % H_2_ and be reversible “on board”. There is a growing doubt in the scientific community that hydride(s) meeting all those requirements would ever be found.

However, in the Hydrogen Economy there would be a number of nonautomotive applications as, for example, portable electronic devices, stationary auxiliary power systems, on-site H_2_ generation systems, off-road vehicles (e.g., forklift trucks), watercrafts, trains and many others that would have much relaxed requirements for fuel cell hydrogen generation and supply system [6]. In the past years, novel hydride systems that are capable of rapid generation of H_2_ at the ambient and low temperatures have been synthesized. So far, we found, that the hydride/hydride [6,7,8,9] and hydride/halide [6,7,8,9] mixtures were capable of mechanical dehydrogenation of about 4 wt % H_2_ after injecting a quite low ball milling energy at the ambient temperature. A few other hydrides also released the quantity of 4 wt % H_2_ although at lower mechanical dehydrogenation rates [6,10,11,12].

In the due course of our experimental work on the hydride systems capable of mechanical dehydrogenation, we recently noticed some proclivity of the hydride/halide (LiAlH_4_-CaCl_2_) system to mechanical dehydrogenation during ball milling (BM) at the ambient temperature. The mechanical dehydrogenation phenomenon has never been investigated for this particular hydride/halide system. The major scientific objective of this work is to investigate and understand in more detail the H_2_ generation behavior from the LiAlH_4_ and CaCl_2_ system in a 2:1 stoichiometric ratio (equivalent to the 1:0.5 ratio) during BM. Thermal dehydrogenation (thermolysis) experiments were also conducted to shed more light on the understanding of the occurring mechanisms.

## 2. Results and Discussion

### 2.1. Morphology of As-Received and Milled Powders

**Figure 1:**
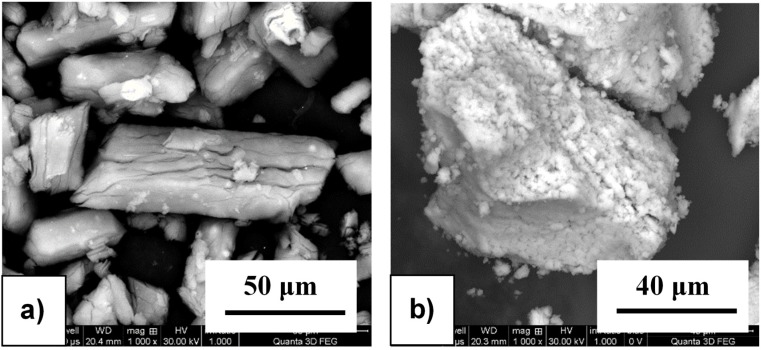
The morphology of as-received powders (**a**) LiAlH_4_ and (**b**) CaCl_2_.

**Figure 2:**
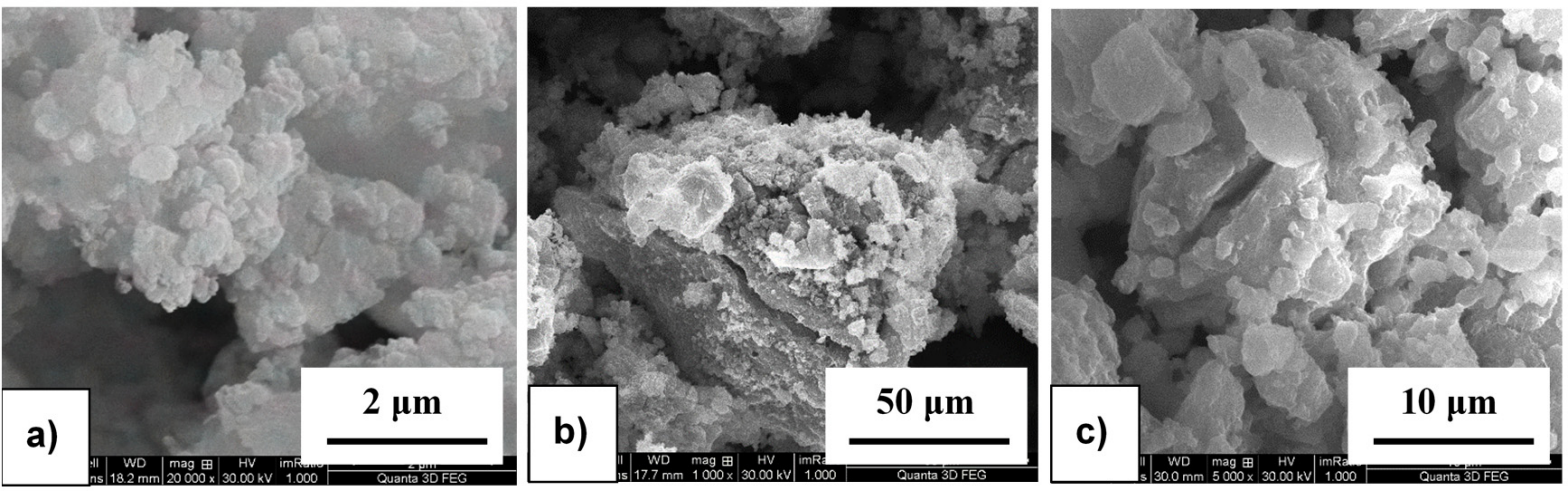
The morphology of the (2LiAlH_4_+CaCl_2_) powders after varying time of milling: (**a**) 5 min; (**b**) 10 min; and (**c**) 5 h.

The morphology of as-received materials and the BM products after different milling times were analyzed with a high resolution scanning electron microscope. The as-received LiAlH_4_ powder is characterized by a cylindrical shape and a non-uniform size distribution with the average length within the 20–60 µm range and the average width within the 10–40 µm range (Figure 1a). In contrast, the average size of the as-received particles of the CaCl_2_ powder is within the 100–200 µm range (Figure 1b). The morphology analysis of milled powders shows a significant particle size reduction even after short milling time for 5 min after which a single particle diameter reaches ~1 µm (Figure 2a). It must be noticed that insufficient milling time causes the presence of unmilled, large particles, nearly 25–50 µm in size (Figure 2b), whereas milling for too long results in a nearly single particle agglomerate formation with the size within the 2.5–5 µm range (Figure 2c).

### 2.2. Microstructural Evolution and Mechanical Dehydrogenation during Ball Milling

**Figure 3:**
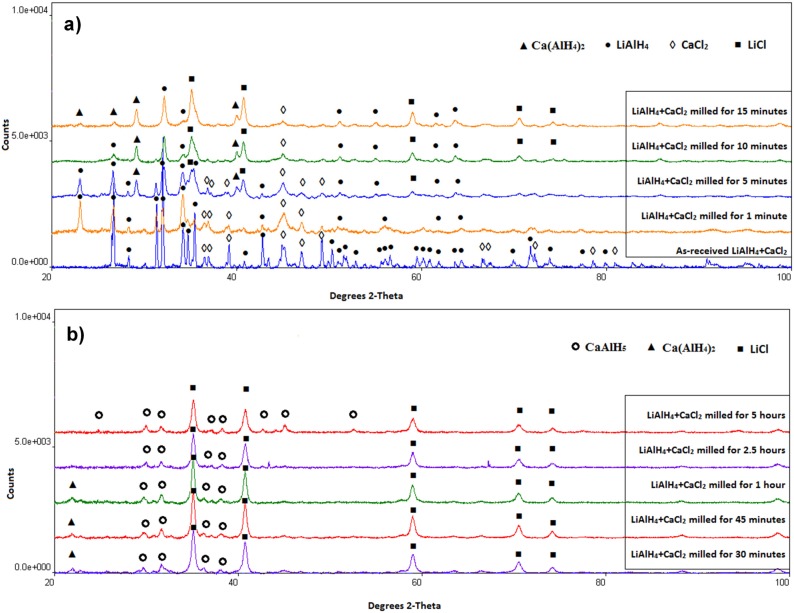
XRD patterns of the (2LiAlH_4_+CaCl_2_) powders after varying milling durations. (**a**) as-received and milling up to 15 min; (**b**) milling from 30 min to 5 h.

The qualitative results of XRD phase analysis for the (LiAlH_4_+CaCl_2_) powders after various milling durations are presented in Figure 3. The XRD patterns for the mixture of as-received reactant powders show the presence of LiAlH_4_ and CaCl_2_. After barely 5 min of BM, the well-recognizable peaks of Ca(AlH_2_)_4_ and LiCl appear on the XRD patterns (Figure 3a). These peaks clearly show that mechano-chemical reaction between these two reactants starts after approximately 5 min of milling. This reaction can described as follows:

2LiAlH_4_+CaCl_2_→Ca(AlH_4_)_2_ + 2LiCl (1)

The theoretical capacity of reaction (1) is 4.32 wt % H_2_ and, accordingly, less taking into account of the practical purity of the reactants. It must be pointed out that reaction (1) was also reported to occur during mechano-chemical activation synthesis (MCAS) of Ca(AlH_4_)_2_ using such reactants as sodium alanate (NaAlH_4_) and calcium chloride (CaCl_2_) [13,14,15,16,17]. So far, the only attempt to use LiAlH_4_ as a reactant with CaCl_2_ to synthesize Ca(AlH_4_)_2_ by MCAS was reported in [13] but without any details.

After 0.5 h of milling (Figure 3b), CaAlH_5_ is also identified which indicates that newly formed Ca(AlH_4_)_2_ starts gradually decomposing during ball milling as a result of mechanical dehydrogenation [6]. The decomposition reaction of Ca(AlH_4_)_2_ could be described as follows:

Ca(AlH_4_)_2_ + 2LiCl→CaAlH_5_ + Al + 1.5H_2_ + [2LiCl] (2)

where the brackets indicate that 2LiCl is not taking part in the reaction being just a dead-weight for the system. The maximum theoretical quantity of H_2_ that could be desorbed in reaction (2) is 1.62 wt % H_2_ (slightly less if the practical purity of the reactants is taken into consideration). Apparently, the decomposition of Ca(AlH_4_)_2_ is associated with mechanical dehydrogenation during ball milling. After 0.5 h (30 min) of milling, only remnant peaks of Ca(AlH_4_)_2_ are discernible in Figure 3b. After 2.5 and 5 h of ball milling no diffraction peaks of Ca(AlH_4_)_2_ are visible anymore (Figure 3b). It must be pointed out that reaction (2) also occurs during *thermal* decomposition of Ca(AlH_4_)_2_ [13,14,15,16,17]. Table 1 summarizes the phases present in the microstructure after various milling durations as established from the XRD analysis. It is to be pointed out that the XRD patterns for powders ball milled from 0.5 to 5 h do not exhibit diffraction peaks of Al whose presence is required by reaction (2). Apparently, newly formed Al in reaction (2) must be in an amorphous or highly nanocrystalline state (undetectable by XRD).

**Table 1:** Summary of phases present in the microstructure after various milling durations as shown in X-ray diffraction (XRD) patterns.

Milling Time	Phases Present
1 min	LiAlH_4_; CaCl_2_
5 min	LiAlH_4_; CaCl_2_; Ca(AlH_4_)_2_; LiCl
10 min	LiAlH_4_; CaCl_2_; Ca(AlH_4_)_2_; LiCl
15 min	LiAlH_4_; CaCl_2_; Ca(AlH_4_)_2_; LiCl
30 min	Ca(AlH_4_)_2_; CaAlH_5_; LiCl
45 min	Ca(AlH_4_)_2_; CaAlH_5_; LiCl
1 h	Ca(AlH_4_)_2_; CaAlH_5_; LiCl
2.5 h	CaAlH_5_; LiCl
5 h	CaAlH_5_; LiCl

### 2.3. Thermal Dehydrogenation

**Figure 4:**
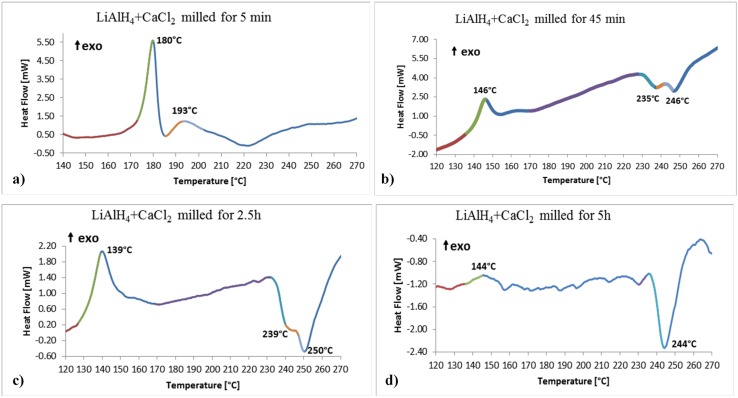
Selected DSC traces for the (2LiAlH_4_+CaCl_2_) powders after varying milling durations. (**a**) 5 min; (**b**) 45 min; (**c**) 2.5 h and (**d**) 5 h. Heating rate 5 °C/min.

Thermal dehydrogenation process of the (2LiAlH_4_+CaCl_2_) ball milled powders was investigated by differential scanning calorimetry (DSC) and thermal gravimetric analysis (TGA). Figure 4 shows the selected examples of DSC curves as a function of ball milling time. For the powder milled for barely 5 min, the first thermal peak is exothermic at around 180 °C (Figure 4a).

This peak temperature correlates very well with temperature for the first exothermic decomposition peak of LiAlH_4_ according to the well-known reaction [3,4]:

LiAlH_4_ → 1/3Li_3_AlH_6_ + 2/3Al + H_2_ (3)

In order to confirm additionally the origin of the first exothermic peak in Figure 4a, we conducted a DSC run for as-received LiAlH_4_ which exhibited a strong exothermic peak with a temperature maximum at 172 °C (a DSC curve not shown here). This confirms well that the first peak in Figure 4a is indeed related to the decomposition process of LiAlH_4_ according to reaction (3). Theoretical capacity of this reaction is 5.3 wt % H_2_ and correspondingly less in the present case because LiAlH_4_ coexists with other phases like CaCl_2_, Ca(AlH_4_)_2_ and LiCl (Table 1), although LiAlH_4_ is still a majority phase in the mixture, which agrees with a high intensity of its DSC peak in Figure 4a. The shape of the LiAlH_4_ decomposition peak suggests that reaction occurs in solid state instead of going through endothermic melting before decomposition as occurs for as-received LiAlH_4_ without additives [18]. The small exothermic peak with the temperature maximum at 193 °C is most likely due to the decomposition of newly formed Ca(AlH_4_)_2_, co-existing in the mixture with the other phases as shown in Table 1. Since Ca(AlH_4_)_2_ is formed in a small quantity after barely 5 min BM, its DSC thermal peak is small, too. It is well known that the first decomposition reaction of Ca(AlH_4_)_2_ according to reaction (2) is exothermic [13,14,15,16,17]. Experimentally observed and reported in the literature DSC peaks for thermal reaction (2) are centered around 150 °C, although on occasions the peak is broad, covering a wide temperature range of 100–160 °C [16,17]. The present peak temperature at 193 °C is slightly higher, perhaps, because it overlaps with the beginning of thermolysis of Li_3_AlH_6_ as discussed below.

The third DSC peak in Figure 4a is *endothermic*, very broad, with the shallow maximum at around 220 °C. It can be assigned to the simultaneous decomposition of both Li_3_AlH_6_ and CaAlH_5_ according to the following reactions [3,4,13,14,15,16,17]:

1/3Li_3_AlH_6_ → LiH + 1/3Al + 0.5H_2_ (4)

CaAlH_5_ → CaH_2_ + Al + 1.5H_2_ (5)

Experimentally observed DSC temperature ranges for reactions (4) and (5) are within 180–260 °C [3,4,18] and 220–270 °C [15,16,17], respectively, which agrees well with thermal events depicted in Figure 4a.

Figure 3a shows that the diffraction intensity of LiAH_4_ peaks decrease after milling for 10 and 15 min because more of them are consumed in reaction (1) forming Ca(AlH_4_)_2_. Accordingly, the obtained DSC curves (not shown here) exhibited a principal exothermic peak having a peak maximum temperature slightly shifted to 172 °C and 158 °C for 10 and 15 min ball milled powders, respectively, which corresponds to the decomposition of retained LiAlH_4_ according to reaction (3). Starting from 30 min milling, there is no LiAlH_4_ in the microstructure (Figure 3b and Table 1) and the exothermic peak of the decomposition of retained Ca(AlH_4_)_2_ in the powder ball milled for 45 min, according to reaction (4), is shifted to ~146 °C (Figure 4b) which is in good agreement with the values reported in the literature being in the range of 100–160 °C [16,17]. The most interesting is a DSC curve in Figure 4c for a powder milled for 2.5 h. It shows pretty strong exothermic peak with the maximum at 139 °C. Apparently, its temperature corresponds very well to the decomposition temperature range of Ca(AlH_4_)_2_. However, the corresponding XRD pattern in Figure 3b shows no peak corresponding to Ca(AlH_4_)_2_. This apparent discrepancy can possibly be explained by proposing a hypothesis that a fraction of Ca(AlH_4_)_2_ mechanically dehydrogenates during ball milling and the other fraction becomes disordered/amorphous, which is undetected by XRD but it still decomposes during DSC measurements. This hypothesis is supported by the shape of a DSC curve for a powder ball milled for 5 h in Figure 4d which shows a small hump at 144 °C most likely corresponding to the decomposition of retained disordered/amorphous Ca(AlH_4_)_2_. Finally, the endothermic DSC peaks in Figure 4b,c, correspond to the decomposition of CaAlH_5_ according to reaction (5).

**Figure 5:**
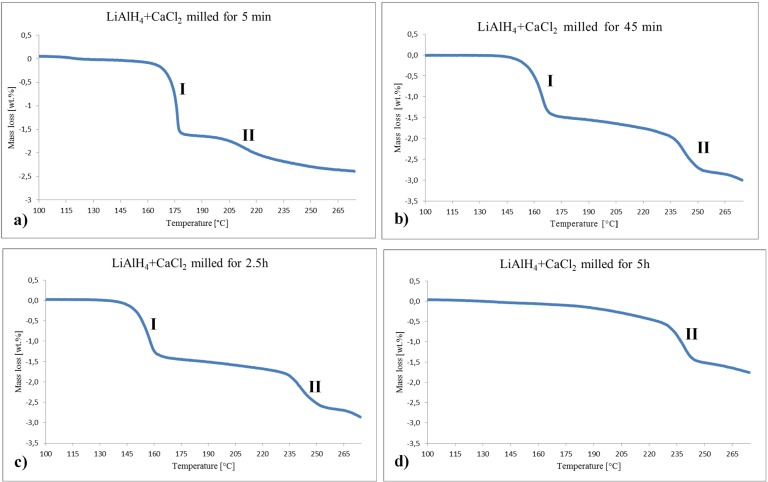
Selected TGA traces for the (2LiAlH_4_+CaCl_2_) powders after various milling durations. (**a**) 5 min; (**b**) 45 min; (**c**) 2.5 h and (**d**) 5 h. Heating rate 5 °C/min.

Figure 5 shows selected curves of TGA, exactly corresponding to the DSC curves in Figure 4. The curves for the powders milled for 5 min, 45 min and 2.5 h exhibit Stage I and II of mass loss. Apparently, Stage I in Figure 5a corresponds to the decomposition of LiAlH_4_ (reaction (3)) and that in Figure 5b,c corresponds to the decomposition of both the crystalline and disordered/amorphous Ca(AlH_4_)_2_ fractions (reaction (2)). There is no clearly visible Stage I in Figure 5d after ball milling for 5 h which confirms that the retained quantity of likely amorphous Ca(AlH_4_)_2_ is nearly negligible. Stage II in Figure 5a corresponds to the decomposition of Li_3_AlH_6_ (reaction (4)) and in Figure 5b–d to the decomposition of CaAlH_5_.

Table 2 summarizes the mass losses in both Stage I and II measured between the inflection points on the TGA curves for each stage and the temperature ranges for both stages. For the 5, 10 and 15 min samples, the values of mass loss are between 1.2 and 1.5 wt %. The observed mass losses correspond to thermolysis of both LiAlH_4_ and Ca(AlH_4_)_2_ (Figure 3a and Table 1), so it is hard to estimate how much of the mass loss is related to the mechanical dehydrogenation of Ca(AlH_4_)_2_ during BM assuming that the observed mass loss in Stage I is fully due to thermal dehydrogenation. However, samples ball milled for 30 min, 45 min and 1 h exhibit thermal mass loss of 1.1, 1.3 and 1.4 wt %, respectively. Assuming that this mass loss directly corresponds to thermal dehydrogenation and the maximum theoretical quantity of mechanically dehydrogenated H_2_ in reaction (2) is 1.62 wt % H_2_, then one can estimate that about 0.5, 0.3 and 0.2 wt % H_2_ was mechanically desorbed during ball milling from samples ball milled for 30 min, 45 min and 1 h, respectively. Therefore, it seems that the mechanical dehydrogenation phenomenon is not quite predominant in the (Ca(AlH_4_)_2_+LiCl) hydride nanocomposite studied in the present work. Furthermore, the sample milled for 2.5 h shows 1.2 wt % mass loss corresponding to 1.2 wt % H_2_ desorbed from this sample in which, most likely, Ca(AlH_4_)_2_ exists as a disordered/amorphous hydride. Accordingly, it shows that about 0.4 wt % H_2_ was mechanically dehydrogenated during ball milling for 5 h, confirming a rather modest occurrence of the mechanical dehydrogenation phenomenon in this study. It is to be pointed out that the mechano-chemical synthesis was carried out in the present work using a Fritsch Pulverisette 7 planetary mill. The milling energy generated by this type of mill is relatively modest by comparison with the magneto-mill Uni-Ball Mill 5 used for inducing a profound mechanical dehydrogenation reported in [3,4,6,7,8,9,10,11,12]. In addition, the milling time up to 5 h used in the present work might have been too short to provide a sufficient milling energy input per gram of powder (kJ/g) to induce more pronounced mechanical dehydrogenation in a Fritsch Pulverisette 7 planetary mill. It would be of interest to repeat mechanical dehydrogenation studies of the present system in the magneto-mill Uni-Ball Mill 5 and compare the results.

Stage II of TGA is related to the decomposition of CaAlH_5_ according to reaction (5) when it is a single phase. However, in the mixture with LiCl reaction (5) is modified to:

CaAlH_5_ + Al + [2LiCl] → CaH_2_ + 2Al + 1.5H_2_ + [2LiCl] (6)

The theoretical capacity of reaction (6) is 1.65 wt % H_2_. Assuming that mass losses for Stage II in Table 2 are due to the hydrogen release; then, approximately only about 50% of the total H_2_ capacity of reaction (6) is dehydrogenated in Stage II. Apparently, CaAlH_5_ seems to be rather stable at a quite elevated temperature range 230–250 °C under a heating rate of 5 °C/min.

**Table 2:** Summary of thermal gravimetric analysis (TGA) mass loss (wt %) at Stage I and II of decomposition of hydride nanocomposites after varying milling time and the temperature ranges of both stages.

Milling Time	Mass loss Stage I (wt %)	Mass Loss Stage II (wt %)	Stage I Range (°C)	Stage II Range (°C)
5 min	1.5	0.4	160–175	205–235
10 min	1.3	0.8	160–175	205–235
15 min	1.2	0.9	145–160	230–250
30 min	1.1	0.7	145–160	230–250
45 min	1.3	0.9	150–170	235–250
1 h	1.4	0.9	147–165	235–250
2.5 h	1.2	0.8	140–160	235–250
5 h	~0.0	1.0	-	230–250

### 2.4. Apparent Activation Energy

**Figure 6:**
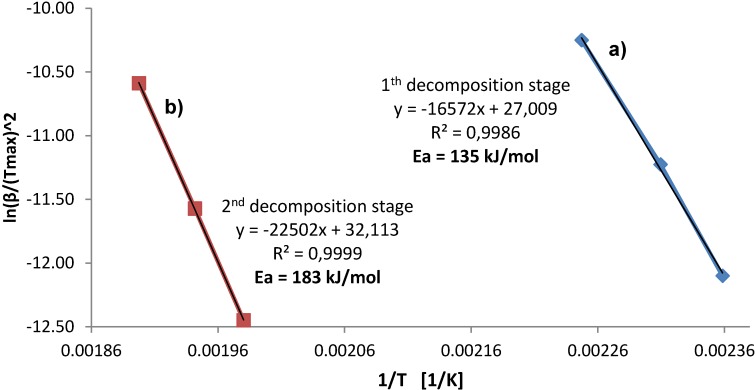
Kissinger plots for (**a**) first and (**b**) second stage of calcium alanate decomposition for the sample milled for 45 min (Figure 4b).

The apparent activation energy, *E*_a_, of decomposition processes occurring in DSC in Figure 4, was estimated in Figure 6 for the sample ball milled for 45 min, by the Kissinger method [19] plotting the maximum reaction rate ln(β/(*T*^2^_max_) as a function of (1/*T*), where β is the heating rate (K/min) and *T*_max_ is the peak temperature in K, carried out with varying heating rates of 1, 2.5 and 7 °C/min for DSC measurements. The reaction rates were obtained from the slopes of the tangent lines at the inflection points of DSC peaks corresponding to H_2_ desorption.

Apparently, a good linearity between ln(β/(*T*^2^_max_) and 1/*T* is obtained with *R*^2^ = 0.99. The apparent activation energy equals 135 kJ/mol and 183 kJ/mol for the first decomposition of Ca(AlH_4_)_2_ and the second decomposition of CaAlH_5_, respectively. Unfortunately, we were unable to find in the literature a value of the apparent activation energy for the Ca(AlH_4_)_2_ decomposition stage obtained in other studies. In contrast, the literature reported values of the apparent activation energy for the CaAlH_5_ decomposition are reported as 161 kJ/mol [20] and 153.4 kJ/mol [16,17]. The value of the apparent activation energy of 183 kJ/mol is close to the reported range although slightly higher. This relatively high value explains the fact that only about 50% of the total H_2_ capacity of reaction (6) is desorbed in Stage II, as discussed earlier, pointing towards an enhanced thermal stability of CaAlH_5_. It is hypothesized that a disordered/amorphous fraction of Ca(AlH_4_)_2_, which likely coexists with CaAlH_5_ after ball milling for 2.5 and 5 h, could be responsible for a sluggishness of CaAlH_5_ thermal decomposition as mentioned earlier.

## 3. Experimental Section

The powders of LiAlH_4_ (99.8% purity, Sigma-Aldrich, St. Louis, MO, USA) and CaCl_2_ (96% purity, Alpha Aesar, Ward Hill, MA, USA) were mixed in the stoichiometric composition 2:1 and ball milled in a Fritsch Pulverisette 7 planetary mill.

Mechano-chemical activation synthesis (MCAS) was carried out for various milling times from 5 min to 5 h in argon gas. The as-received powder mixture was loaded with ten stainless-steel balls, 10 mm in diameter, into 20 mL bowl made of hardened-steel. The ball-to-powder weight ratio was 40:1 and the rotational speed of the milling bowl was 650 rpm. The powder, before and after milling, was kept in a Labmaster Workstation (MBraun Inert-Gas Systeme GmbH, Garching, Germany) under continuously purified argon atmosphere with <0.1 ppm O_2_ and H_2_O vapor. The samples after 5, 10, 15, 30, 45 min and 1, 2.5 and 5 h of milling were taken for further study.

Morphological examination of powders was conducted with high-resolution SEM Quanta 3D FEG Dual Beam (Quanta, Hillsboro, OR, USA). The samples of loose powder were attached to a carbon tape and coated by gold in vacuum.

The phase composition of the samples was checked by X-ray diffraction (XRD) on Rigaku ULTIMA IV (Rigaku Corporation, Tokyo, Japan) using a CuK_α1_ radiation, generated at an accelerating voltage of 40 kV and a current of 40 mA. To prevent samples, oxidation and hydrolysis tested samples were sealed in glass capillaries. The scan range was from 2θ = 20°–120° and the scan rate was 0.6°·min^−1^. The X-ray profiles were analyzed with the PDXL software (Rigaku, Tokyo, Japan) working with a PDF-4 database. The following ICDD (JCPDS) file numbers were used for phase identification: CaCl_2_-12-0056; Ca(AlH_4_)_2_-14-5077; LiCl-04-0664; CaAlH_5_-14-5080.

The thermal behavior of powders was studied by Differential Scanning Calorimetry (DSC) (LabSys-SETARAM, SETARAM Instrumentation, Caluire, France) using 5–10 mg samples with heating rate of 5 °C/min.

Mass loss of samples was measured by TGA using LabSys-SETARAM and estimated with respect to the total mass of powder. The samples with the 120–160 mg weight were tested with heating rate 5 °C/min and in the temperature range of 50–270 °C.

## 4. Conclusions

A hydride composite of Ca(AlH_4_)_2_ and LiCl was synthesized using mechano-chemical activation synthesis (MCAS) employing LiAlH_4_ and CaCl_2_ as the reactants, during relatively short milling duration. Ca(AlH_4_)_2_ starts decomposing into the mixture of CaAlH_5_+2Al+1.5H_2_ during ball milling for at least 0.5 h and longer which is evidenced by the CaAlH_5_ diffraction peaks appearing on XRD patterns. Mechanical dehydrogenation not exceeding 1 wt % H_2_ is associated with the Ca(AlH_4_)_2_ decomposition during ball milling in a Fritsch Pulverisette 7 planetary mill up to 2.5 h. After 2.5 h of ball milling no Ca(AlH_4_)_2_ diffraction peaks were observed on XRD patterns which might suggest that Ca(AlH_4_)_2_ was fully decomposed. On the other hand, thermal behavior of ball milled powders, which was investigated by thermal gravimetric analysis (TGA) and differential scanning calorimetry (DSC), indicated that a certain fraction of Ca(AlH_4_)_2_ was disordered/amorphized during ball milling. The apparent activation energy of 135 kJ/mol and 183 kJ/mol was estimated for the thermolysis of Ca(AlH_4_)_2_ and CaAlH_5_, respectively, both co-existing with LiCl in a composite.
